# Thrombocytopenia Following Isolated Surgical Aortic Valve Replacement With Inspiris Resilia Bioprosthesis

**DOI:** 10.37825/2239-9747.1065

**Published:** 2024-12-04

**Authors:** Michele D’Alonzo, Lorenzo Di Bacco, Massimo Baudo, Antonella D’Alonzo, Yudit Tesfaye Dossena, Francesco Rattenni, Claudio Muneretto, Fabrizio Rosati

**Affiliations:** a Division of Cardiac Surgery, Spedali Civili Hospital, University of Brescia, Brescia, 25124, Italy; b Division of Cardiac Surgery, Henri Mondor Hospital, Creteil, 9400, France; c Department of Cardiac Surgery Research, Lankenau Institute for Medical Research, Main Line Health, Wynnewood, PA, 19096, USA; d Medicine and Surgery School, University of Roma “La Sapienza”, Roma, 00155, Italy; e Department of Cardiology and Cardio-Vascular Surgery, Haut-Leveque Hospital, Pessac, 33600, France

**Keywords:** Thrombocytopenia, Aortic valve replacement, Inspiris Resilia, Carpentier Magna Ease, Platelet count reduction

## Abstract

**Aims:**

Thrombocytopenia (TCP) following aortic valve replacement (AVR) is a significant concern, with varying degrees of platelet count reduction observed postoperatively. This phenomenon occurs more frequently in patients undergoing surgical biological AVR compared to those receiving mechanical AVR or transcatheter aortic valve implantation (TAVI). This study aimed to investigate the incidence of TCP following surgical AVR with the Inspiris Resilia bioprosthesis compared with Carpentier Magna Ease valve.

**Methods:**

The study retrospectively collected data from 144 patients who underwent isolated AVR between January and December 2023. Patients received either the Inspiris Resilia or the Carpentier Magna Ease valve. Platelet counts were evaluated from admission to discharge, and statistical analyses were performed to identify significant variables correlated to post-operative TCP.

**Results:**

Patients receiving the Inspiris Resilia valve had higher platelet counts from postoperative day 1–3 compared to those with the Carpentier Magna Ease valve. The incidence of moderate-to-severe TCP was significantly lower in the Inspiris Resilia group. However, no significant differences in clinical outcomes, such as bleeding events and blood transfusion rates, were observed between the two groups.

**Conclusions:**

The study showed that the Inspiris Resilia valve is associated with a lower incidence of moderate-to-severe TCP compared to the Carpentier Magna Ease valve. These findings underscore the potential benefits of utilizing the Inspiris Resilia valve to mitigate the risk of TCP post-AVR. Further research with larger cohorts is warranted to validate these results and explore the underlying mechanisms.

## 1. Introduction

Thrombocytopenia (TCP) following surgical aortic valve replacement (sAVR) with bioprostheses remains a matter of debate. Various degrees of transient postoperative platelet (PLT) count reduction have been observed, irrespective of the type and generation of biological surgical valves [1].

Recently, this phenomenon has also been noted with the use of the latest Sutureless, Rapid deployment devices, and transcatheter aortic bioprostheses [2]. Indeed, this recent meta-analysis identified TCP as an independent predictor of poorer outcomes for patients undergoing transcatheter aortic valve implantation (TAVI). However, the frequency of TCP phenomenon is higher in patients who have undergone cardiac surgery compared to those who received TAVI [3]. Additionally, the same research indicated that certain surgical bioprosthetic models are more prone to thrombocytopenia, with the duration of cardiopulmonary bypass emerging as a critical factor in the onset of postoperative thrombocytopenia.

The mechanisms underlying TCP are not fully understood. Widely accepted hypotheses include PLT activation resulting from interaction with bioprosthetic leaflets and PLT destruction induced by shear stress leaded during cardiopulmonary bypass. Not only, the mechanisms causing low PLT count after cardiac surgery include changes in microcirculatory status, immune-mediated processes and the influence of heparin [4–6].

Lastly, the chemical components used for pericardium stabilization during leaflet manufacturing may influence PLT function [7].

Previous studies have shown that bioprostheses stored in glutaraldehyde-based solutions are associated with a lower degree of TCP compared to those stored in homocysteic acid [8].

In this context, the bovine pericardial Inspiris Resilia aortic valve (Edwards Lifesciences, Irvine, USA) represents the latest evolution of bioprostheses, featuring an innovative pericardial treatment and storage method. The pericardium of this valve is treated with a novel integrity preservation method that permanently blocks calcium binding sites, allowing for dry storage without the need for rinsing before use. At five years of follow-up, excellent hemodynamic performance and freedom from structural valve deterioration, along with a favourable safety profile, have been reported [9]. However, data regarding the impact of this valve on postoperative TCP are limited.

We aimed to investigate the incidence of TCP following sAVR with the Inspiris Resilia aortic valve and to compare this novel bioprosthesis with a standard generation bovine bioprosthesis, the Carpentier Magna Ease (Edwards Lifesciences, Irvine, CA).

## 2. Methods

Data of 144 consecutive patients undergoing isolated aortic valve replacement by means of either Inspiris Resilia (IR) or Carpentier Magna Ease (CME) bioprostheses between January and December 2023 were retrospectively collected in our institution. The bioprosthesis was chosen at the surgeon’s discretion. Exclusion criteria were any combined AVR surgery, reintervention, acute endocarditis or septic shock, preoperative <100.000/mm3 PLTs, hematologic malignancies and history of heparin induced thrombocytopenia.

The study was conducted according to the guidelines of the Declaration of Helsinki and approved by the Institutional Review Board of University of Brescia (protocol code NP 2405 - named TPS003 - date of approval 21/07/2016).

Data about PLTs count were retrieved from institutional informatic systems. Platelet count was evaluated at the time of admission, during the first four days post-operatively and at discharge. Thrombocytopenia was defined as PLTs count <150.000/mm3 with the following criteria: mild TCP (100–149.999/mm3), moderate TCP (50–99.999/mm3), severe TCP (<50.000/mm3) while a normal count was considered for PLTs ≥150.000/mm3.

Acetylsalicylic acid was not routinely discontinued, while oral anticoagulants were stopped according to institutional protocol 48 hours before admission and low molecular weight heparin (LMWH) started after coagulation dosage control. LMWH was not administered the day of the index procedure. A 300 UI/kg dose of heparin was administered for systemic heparinization for CPB conduction. At the end of the procedure, heparin was antagonized using 1–1.5 mg of protamine solfate every 100 UI of heparin. During the surgical procedure, a cell salvage machine was used as per internal Institutional protocol. As a standard of care, all patients started antithrombotic prophylaxis after sAVR with subcutaneous Low Molecular Weight Heparin (LMWH) administered until postoperative day 3. If the patient did not have any clinical indications for full-dose anticoagulation (e.g., atrial fibrillation), LMWH was discontinued, and the patient was treated only with acetylsalicylic acid (100 mg orally, daily). Otherwise the patient was treated only with warfarin for a period of 3 months as recommended by current European guidelines [10]. In any case, at our institution acetylsalicylic acid was started within the first two days after surgery.

### 2.1. Endpoints

The primary outcomes aimed to evaluate the incidence of thrombocytopenia following cardiac surgery, differentiated by the type of biological valve utilized (CME or IR). This evaluation was conducted over a daily timeframe (from postoperative day 1 to discharge). Also, the severity of thrombocytopenia was categorized according to previously defined criteria. Additionally, the nadir for each patient group was calculated. The nadir was defined as the lowest point reached during hospitalization. The maximum drop was calculated by the differences between pre-operative count and the nadir.

The secondary outcomes focused on the clinical implications of thrombocytopenia. These included need of transfusion, occurrence of major bleeding events, measurement of blood loss observed through the surgical drains within 48 hours from the cardiac surgery procedure.

Finally, the nadir was correlated with a traditional risk factor for thrombocytopenia, specifically with the duration of extracorporeal circulation.

### 2.2. Statistical analysis

Categorical variables were reported as frequency (%). Continuous variables were presented as mean ± standard deviation (SD) or median [inter-quartile range (IQR)]. All continuous variables were checked for normality using the Kolmogorov-Smirnov test. Comparisons were made using a Pearson’s Chi-Square or Fisher’s exact test for categorical variables, accordingly. For continuous variables comparisons were performed according to their distribution, using a Student’s t-test or Mann-Whitney-U test as needed.

A logistic regression model was employed to identify a priori factors correlated to the incidence of moderate-to-severe TCP. An univariable model was used for initial variable selection. Only variables with a p-value of <0.2 were included in the multivariable regression model. The effect size of the variables was estimated by calculating the odds ratio (OR) and 95 % confidence interval (CI). Correlations between post-operative PLT nadir and traditional risk factors of thrombocytopenia were calculated using Pearson’s correlation.

A p-value of <0.05 was considered statistically significant. Microsoft Office Excel (Microsoft, Redmond, Washington) was used for data extraction and management, while all analyses were performed in R version 4.3.1 (R Software for Statistical Computing, Vienna, Austria) within RStudio. Used packages included “lattice”, “ggplot2”, “RColor-Brewer”, “gtsummary”.

## 3. Results

Seventy patients received the Inspiris Resilia aortic valve, while 74 patients received the Carpentier Magna Ease aortic valve. Patients’ baseline characteristics are listed in Table S1 (Supplementary material) (Translational Medicine@UniSa:EdiKit). Patients in the CME group were older when compared to IR patients (IR 65.2 ± 7.1 years old vs CME 68.5 ± 8.3 years old, p: 0.009) with comparable risk assessment. Echocardiography revealed a higher percentage of patients with bicuspid aortic valve treated by Inspiris Resilia implantation (IR: 48.6 % vs CME: 23.0 %, p: 0.001).

**Fig. 1:**
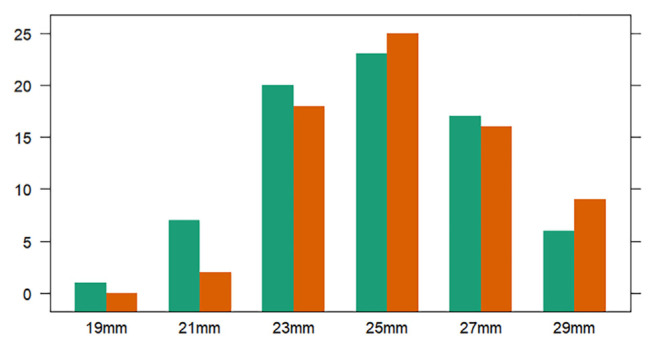
Valve size implantation frequency. P value: 0.481.

Operative data are depicted in Table S2 (Supplementary material) (Translational Medicine@UniSa: EdiKit). Minimally invasive approach (partial sternotomy) was used more frequently in the IR group (IR: 60 % vs CME: 40.5 %, p: 0.02). Comparable times for aortic cross clamp and cardiopulmonary bypass time were founded between the two groups. Surgeons with a strong preference for the Carpentier Magna Ease often adopt an interrupted suture technique for implantation. No differences in terms of valve size were found between the two group (Fig. 1, p value: 0.481).

**Fig. 2:**
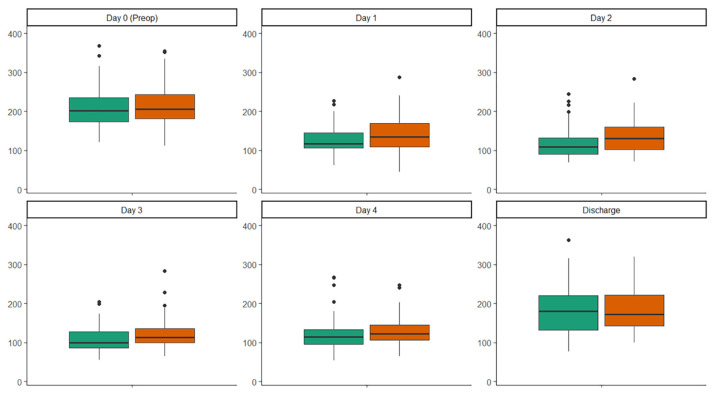
Box plot depicting PLT count at different timeframe according to bioprosthesis implanted.

In Fig. 2 and Table 1 show the platelet (PLT) count analysis. At admission pre-operative PLTs count was similar between groups (p: 0.569). Moreover, CME group showed a significantly lower median PLTs count at any time from post operative day 1 to post operative day 3 (p: 0.021, 0.004, 0.022, respectively), while after this timeframe the difference in the two group did not reach statistical significance (p: 0.142 and p: 0.790 at day 4 et at discharge, respectively).

No differences were reported in terms of incidence of PLTs level <150.000/mm3 at least once in any postoperative measurement between the two groups (IR: 85.7 % vs CME: 92.3 %, p: 0.140) but considering moderate-to-severe TCP (<100.000 PLT/mm3) the IR group was less susceptible to this phenomenon (IR: 37.2 % vs CME: 63.5 %, p: 0.001). The median nadir was lower in the CME group (p: 0.004) and also the maximum drop was more severe in the CME group (p: 0.006).

The secondary endpoints revealed no differences in terms of clinical outcomes concerning the postoperative thrombocytopenia as depicted in Table 2. The rate of blood transfusion was comparable between the two groups (IR: 41.4 % vs CME: 50.0 %, p: 0.302) with similar blood loss from chest drainages (IR: 550.1 ± 142.7 mL vs CME: 559.5 ± 140.1 mL, p: 0.344). The incidence of major bleeding was comparable either considering the rate of surgical revision required or considering the adverse events such as cerebral or gastric haemorrhage or melena. There is only a patient who died during the main hospitalization due to severe low cardiac output syndrome in the first postoperative day. No significant paravalvular leaks were founded at discharge with length of stay resulting comparable between the two groups (p: 0.895).

Table 3 presents the a priori variables correlated to the incidence of moderate-to-severe thrombocytopenia, defined as at least one postoperative measurement of <100,000 platelets/mm^3^. In the multivariable analysis, two factors were found to be correlated to this outcome: cardiopulmonary bypass time showed a significant association (OR = 1.02, 95 % CI: 1.00–1.04, p = 0.010), indicating that longer extracorporeal circulation were linked to increased risk. The use of the Carpentier Magna Ease bioprosthesis was also significantly associated with the outcome (OR = 3.50, 95 % CI: 1.61–7.59, p = 0.002), suggesting a higher likelihood of the event occurring compared to other bioprostheses. Conversely, other factors, including bicuspid aortic valve morphology and full sternotomy surgical, did not show any significant associations with the outcome in the multivariable model.

Figure S1 (supplementary material) (Translational Medicine@UniSa:EdiKit) shows a very weak correlation between the maximum PLT drop and the length of extracorporeal circulation: for Inspiris Resilia group, Pearson coefficient R is −0.046 (R^2^ 0.0021, p value: 0.705). For Carpentier Magna Ease R: −0.192 (R^2^: 0.0369, p value: 0.101).

## 4. Discussion

This study focused on the effect on postoperative TCP related to the use of a new generation aortic bioprosthesis with a peculiar pericardial treatment and storage, namely Inspiris Resilia, compared to the standard model Carpentier Magna Ease.

The main findings of this study are summarized as follows:

- Patients receiving IR had superior platelet count from postoperative day 1 to postoperative day 3 compared to CME.
- The use of CME was significantly associated with moderate to severe post-operative thrombocytopenia.
- In this series postoperative TCP did not affect clinical outcomes in patients receiving both IR and CME valves.

Different degrees of platelet count reduction are reported after sAVR, irrespective of the bioprosthesis type—stented, stentless, sutureless or rapid deployment valves [7,11]. The authors also demonstrated that aortic bioprostheses led to a more severe decrease in platelet count compared to other cardiac surgery operations with similar CPB times, such as coronary artery bypass grafting (CABG) or mechanical sAVR, suggesting that shear forces and chemical washout may play a role in this phenomenon.

Vogt et al. found that some surgical bioprosthetic models, particularly stentless and rapid deployment valves, are more susceptible to platelet drop than others, which may be related to specific pericardial tissue treatments and to the presence of exposed nitinol stent support in the blood flow [3]. A recent randomized controlled trial reported that the Perceval Sutureless aortic bioprosthesis was associated with a higher rate of transient platelet reduction compared to stented bioprostheses, though this reduction was transient and clinically non-relevant [12].

In this study, a trend of platelet count reduction in both groups was noted, consistent with previous reports and meta-analyses [1,2,7,13]. Although the trend was present for both bioprostheses generation, was more pronounced in the CME group on postoperative days 1, 2, and 3. However, in the following hospitalization days the difference between the two groups became negligible. The nadir of platelet reduction occurred at POD 3 for both groups. This finding aligns with other studies reporting nadirs between POD 2 and POD 3 [7,8].

Given the multifaceted nature of TCP, a unanimous consensus on its mechanisms is still lacking to date. Potential causes include abnormal platelet activation due to interaction with chemically treated pericardial leaflets, endothelial damage, and turbulent flow from prosthesis implantation [14]. Hystorically, pericardial treatment was indicated as the main cause of such complication in stentless bioprostheses, treated with homocysteic acid and stored in aldehyde-free solutions showing a higher predisposition to TCP [1]. This aberrant activation might be caused by several triggers: interaction of circulating blood with chemically treated pericardial leaflets used for tissue preservation and storage (i.e., glutaraldehyde and homocysteic acid), endothelial damage and shear stress modifications caused by prosthesis implantation, or turbulent annular flow due to the presence of residual tissue protruding at the level of the inflow side.

In spite of this IR bioprosthesis undergoes a novel processing technology that avoids aldehyde-derived solutions, potentially reducing the risk of platelet activation and TCP. In contrast, the CE Magna Ease utilizes the ThermaFix Process, which employs low-pressure fixation to preserve collagen elasticity and reduce aldehyde residues [15]. This difference in pericardial chemical treatment methods might explain the observed differences in TCP between the two valve types.

Moreover, this study pointed out that use of cardiopulmonary bypass (CPB) is a significant factor contributing to transient postoperative TCP, with CPB duration being a major determinant in patients with prologend CPB time [3]. Our study aimed to identify variables correlated to moderate-to-severe TCP, defined as postoperative platelet counts below 100,000/mm^3^. Logistic regression analysis identified CPB duration and the use of Carpentier Magna Ease bioprosthesis to be significantly associated to TCP, whereas age, implantation techniques, and surgical approach were not. Nevertheless, the fact that CPB serves as a predictor of at least one instance of TCP does not affect its severity: this was demonstrated by the minimal correlation between the maximum platelet decrease and the duration of extracorporeal circulation in both bioprostheses, as illustrated in Figure S1 (Translational Medicine@UniSa:EdiKit).

In addition, the implantation technique may represent an important factor deserving further investigation. It has been hypothesized that the suturing method (continuous versus interrupted with pledgets) could impact valve hemodynamic and performance, possibly playing a role in the aetiology of postoperative thrombocytopenia. Interrupted sutures in aortic valve replacement can create slight irregularities along the suture line, potentially leading to uneven valve seating. This may result in mild narrowing of the left ventricular outflow tract (LVOT) and increased turbulence in blood flow. Turbulent flow elevates shear stress on platelets, which can lead to their activation and consumption. This process, known as shear-induced platelet activation, may result in platelet aggregation and subsequent depletion, contributing to thrombocytopenia. While the correlation between the continuous suture technique in aortic valve replacement (AVR) and postoperative thrombocytopenia is not fully established, continuous suturing creates a smooth, uniform suture line, which may reduce turbulence and shear stress compared to interrupted sutures. However, continuous sutures result in longer suture lines, potentially exposing a larger area to blood flow and increasing the chance of platelet activation as blood interacts with the suture material. This could lead to platelet consumption and contribute to postoperative thrombocytopenia. Suturing techniques are more frequently correlated with other postoperative outcomes, such as paravalvular leak or pacemaker requirement [16], while thrombocytopenia is more closely linked to valve type, operative/CPB duration, haemodilution and RBC transfusion. As a matter of fact, our logistic regression analysis revealed no significant correlation between suturing technique and thrombocytopenia (Table 3).

Several studies have suggested that the mechanism involved in platelet (PLT) reduction is related to hemodynamic turbulence caused by inaccurate valve sizing, particularly with the implantation of small prostheses, as well as by manipulation of the bioprosthesis before deployment. However, in the present study, although the two patient groups had comparable prosthesis sizes and similar designs (Fig. 1), the CME group experienced a significantly lower platelet count in the postoperative period. This suggests that multiple factors are interplaying in the aetiology of thrombocytopenia, including patient-specific factors and operative variables. Moreover, in this scenario valve type and pericardial treatment played an important role.

Significant platelet count reduction is also observed after transcatheter aortic valve implantation (TAVI). In this subpopulation thrombocytopenia following TAVI with the Edwards’ Sapien valves is a frequent but generally self-limited process without clinical consequences [17].

Postoperative TCP’s clinical implications vary among reported papers. Some studies link severe TCP with higher rates of complications, such as bleeding and acute kidney injury [18,19], while others report no significant differences in early mortality or neurological outcomes between valve types despite different TCP rates [13,20]. Our study found no significant differences in clinical outcomes related to TCP between the IR and CME groups, with comparable rates of blood transfusion and similar blood loss from chest drains.

### 4.1. Limitations

One of the limitations of our study is the small sample size and the retrospective nature of the analysis with all the related biases. However, despite the limitations of the current study and the nonuniform nature of the existing published data, we believe that having over 70 patients in each group with comparable baseline characteristics provides sufficient strength to draw preliminary conclusions. Therefore, more powerful studies are warranted to confirm these results. Finally, no comprehensive haematological studies were conducted on patients who exhibited severe thrombocytopenia.

### 4.2. Future directions

Thrombocytopenia in haematological patients undergoing aortic valve replacement requires careful management to minimize the risk of bleeding complications and ensure optimal surgical outcomes. This involves a multidisciplinary approach, including the careful selection of analgesia and anticoagulation strategies, as well as close monitoring of preoperative and postoperative outcomes. Additionally, prosthesis selection proves to be crucial; for patients undergoing aortic valve surgery, the choice of the Inspiris Resilia prosthesis may be considered due to its lower levels of postoperative thrombocytopenia.

## 5. Conclusions

Thrombocytopenia following bioprosthetic aortic valve replacement is a frequent and multifactorial phenomenon, though its aetiology remains uncertain. While this study showed a significant but transient reduction in platelet count associated with the CME valve compared to the IR valve, no differences in clinically relevant adverse events.

## Figures and Tables

**Table 1:** Platelets count analysis.

	OVERALL POPULATION (n = 144)	INSPIRIS RESILIA (n = 70)	CARPENTIER MAGNA EASE (n = 74)	p value
Day 0 (Pre-op)	205 (177–238)	206 (181–243)	203 (175–235)	0.569
Day 1	127 (106–159)	135 (109–169)	117 (106–145)	**0.021**
Day 2	117 (91–150)	131 (102–160)	110 (90–132)	**0.004**
Day 3	110 (90–131)	113 (99–135)	100 (86–128)	**0.022**
Day 4	119 (100–143)	122 (106–145)	115 (95–134)	0.142
At discharge	173 (134–222)	172 (143–223)	180 (131–220)	0.790
Nadir	100 (80–124)	108 (91–133)	90 (74–117)	**0.004**
Maximum drop	−103 (126–79)	−98 (122–71)	−107 (129–85)	**0.006**
PLT <150 *	129 (89.6)	60 (85.7)	69 (93.2)	0.140
PLT <100 *	73 (50.7)	26 (37.2)	47 (63.5)	**0.001**

Values are expression in events (%) or median (quartile 1–3). PLT: platelets (count *1000/mm3).

* at least in one measurement post op.

**Table 2:** Post-operative complications.

	OVERALL POPULATION (n = 144)	INSPIRIS RESILIA (n = 70)	CARPENTIER MAGNA EASE (n = 74)	p value
In-hospital mortality	1 (0.7)	1 (1.4)	0	0.486
Post-operative AF	53 (36.8)	24 (34.3)	29 (39.2)	0.542
PVL	0	0	0	–
AKI requiring CVVH	6 (4.4)	2 (2.9)	4 (6.1)	0.431
Bleeding requiring surgical revision	5 (3.5)	2 (2.9)	3 (4.1)	0.999
Non thoracic bleeding (melena, haemorrhagic cerebral, gastrointestinal)	4 (2.8)	2 (2.9)	2 (2.7)	0.999
Blood transfusion	66 (45.8)	29 (41.4)	37 (50.0)	0.302
Chest tube loss (mL)	554.9 ± 141.0	550.1 ± 142.7	559.5 ± 140.1	0.344
Length of stay (days)	7 (6–8)	7 (6–8)	7 (6–8)	0.895

Values are expression in frequency (%) and median (quartile 1–3). AF: atrial fibrillation; PVL: paravalvular leak; AKI: acute kidney injury; CVVH: Continuous veno-venous hemofiltration.

**Table 3:** Logistic regression determinant a priori variables correlated to moderate-to-severe platelet count (defined as <100.000 PLT/mm3 at least in one measurement after surgery).

	Univariable model			Multivariable model		
	
	OR	95 % CI	p value	OR	95 % CI	p value
Age	1.02	0.98–1.06	0.444			
CPB	1.01	1.00–1.03	0.035	1.02	1.00–1.04	0.010
Gender (male)	1.50	0.71–3.18	0.294			
AV morphology (bicuspid)	1.60	0.80–3.19	0.180	1.24	0.58–2.66	0.574
Implantation technique (continue suture)	0.75	0.39–1.45	0.392			
Bioprosthesis (Carpentier Magna Ease)	2.95	1.50–5.81	0.002	3.50	1.61–7.59	0.002
Surgical approach (full sternotomy)	1.85	0.96–3.59	0.068	1.26	0.61–2.58	0.535

OR: odd ratio; CI: confident interval; CPB: cardiopulmonary bypass; AV: aortic valve.

## Data Availability

The raw data supporting the conclusions of this article will be made available by the authors on request.
